# Graft and patient survival following angioplasty for post-transplant portal vein stenosis in children under 7 years: a matched case–control study

**DOI:** 10.1186/s42155-025-00619-x

**Published:** 2025-12-13

**Authors:** Simone Hammer, Anna Dorn, Michael Christian Doppler, Florian Zeman, Christian Stroszczynski, Dirk Grothues, Birgit Knoppke, Stefan M. Brunner, Hans Jürgen Schlitt, Wibke Uller

**Affiliations:** 1 Department of Radiology, Medical Center, University of Regensburg, Faculty of Medicine, Regensburg, 93053 Germany; 2 Department of Diagnostic and Interventional Radiology, Medical Center, University of Freiburg, Faculty of Medicine, University of Freiburg, Hugstetterstr. 55, Freiburg, 79106 Germany; 3 Center for Clinical Studies, Medical Center, University of Regensburg, Faculty of Medicine, Regensburg, 93053 Germany; 4 University Children’s Hospital Regensburg (KUNO), Medical Center—University of Regensburg, Faculty of Medicine, Regensburg, 93053 Germany; 5 Department of Surgery, Medical Center, University of Regensburg, Faculty of Medicine, Regensburg, 93053 Germany

**Keywords:** Liver transplantation, Portal vein, Stenosis, Angioplasty, Stent, Graft survival

## Abstract

**Background:**

Portal vein (PV) stenosis (PVS) is a common issue after pediatric liver transplantation (LT) and may be associated with severe morbidity. The purpose of this retrospective matched cohort study was to compare graft and patient survival after percutaneous angioplasty (PTA) for PVS in pediatric patients under 7 years with a control cohort without PVS and a cohort with chronic portal vein thrombosis (PVT).

**Methods:**

This study included 31 patients with PVS (intervention group) and 62 patients without PVS (control group). Furthermore, 9 patients with chronic PVT were evaluated (negative control group). Primary endpoints were graft and patient survival. Secondary endpoints were comparison of liver function and clinical course (signs of portal hypertension), procedure-related complications, and long-term patency after PTA.

**Results:**

Graft survival was comparable between the intervention and control group (*p* = 0.380), with 7-year survival rates of 93% (95% confidence interval [CI]: 84%, 100%) and 88% (95% CI: 80%, 97%), respectively. Graft survival was significantly higher in the intervention group compared to the negative control group *p* = 0.032; 7-year graft survival was 75% (95% CI: 50%, 100%). Patient survival was significantly higher in the intervention group (*p* = 0.034), with a 100% 7-year survival rate (95% CI: 100%, 100%) compared to 86% (95% CI: 78%, 96%) in the control group. Survival was reduced in the negative control group compared to the intervention group (*p* = 0.053). Seven-year patient survival in the negative control group was 88% (95% CI: 67%, 100%). There was one minor procedure-related complication (1/31 [3%]); long-term patency was 100%.

**Conclusions:**

Seven-year graft and patient survival after PTA for PVS were on par with that of patients without PVS. Graft survival was lower for patients with chronic PVT. Clinical course and liver function after PTA were comparable to patients without PVS. These findings, together with the low complication rate and high long-term patency, provide additional evidence supporting the efficacy of PTA in the management of PVS.

**Graphical Abstract:**

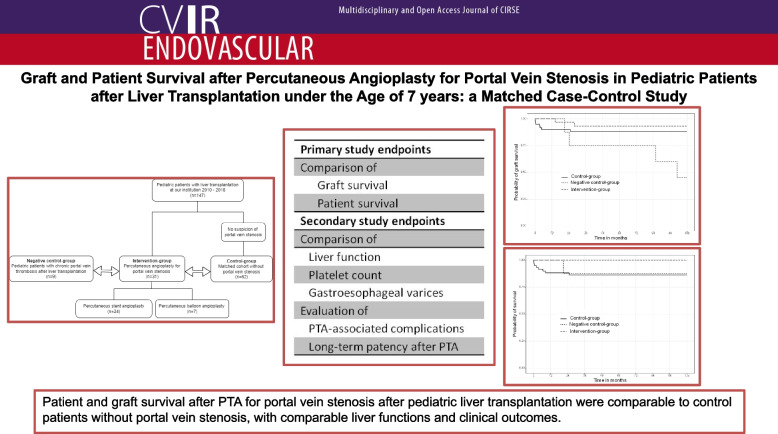

## Background

Liver transplantation (LT) is the standard care for pediatric patients with end-stage liver disease [1]. Despite advancements in surgical techniques, portal vein (PV) complications (PVC) like PV stenosis (PVS) and thrombosis (PVT) remain frequent with a reported incidence ranging from 8 to 16% [2–4]. The clinical course of PVS ranges from asymptomatic to severe portal hypertension (PH) like gastrointestinal variceal bleeding. Active monitoring is crucial for the early detection and successful management of PVS [5].

Treatment options for PVS after LT include conservative therapy, endovascular interventions, and surgical procedures such as the mesorex bypass [6]. The systematic review of Alfares et al. demonstrated the efficacy and safety of percutaneous angioplasty (PTA) with long-term primary patency rates ranging from 75 to 94% [5]. The recent study of Messana et al. reports a long-term patient survival rate of 96% after PTA for PVS [7]. However, the effect of successful PTA on long-term graft survival and patient outcomes in comparison to matched control patients remains unexplored.

The primary aim of this study was to compare graft and patient survival after percutaneous treatment of PVS in children after LT under the age of 7 years with those of a matched control group without PVS and those with chronic PVT (negative control group). The secondary objectives were (1) to compare posttreatment liver function and clinical outcomes with those of the control group and negative control group, (2) to compare pre- with posttreatment signs of PH, and (3) to report treatment-associated complications and long-term patency.

## Methods

Institutional review board approval was obtained. The need for written informed consent for study participation was waived due to the retrospective design.

### Study design and selection of the study cohorts

**Fig. 1:**
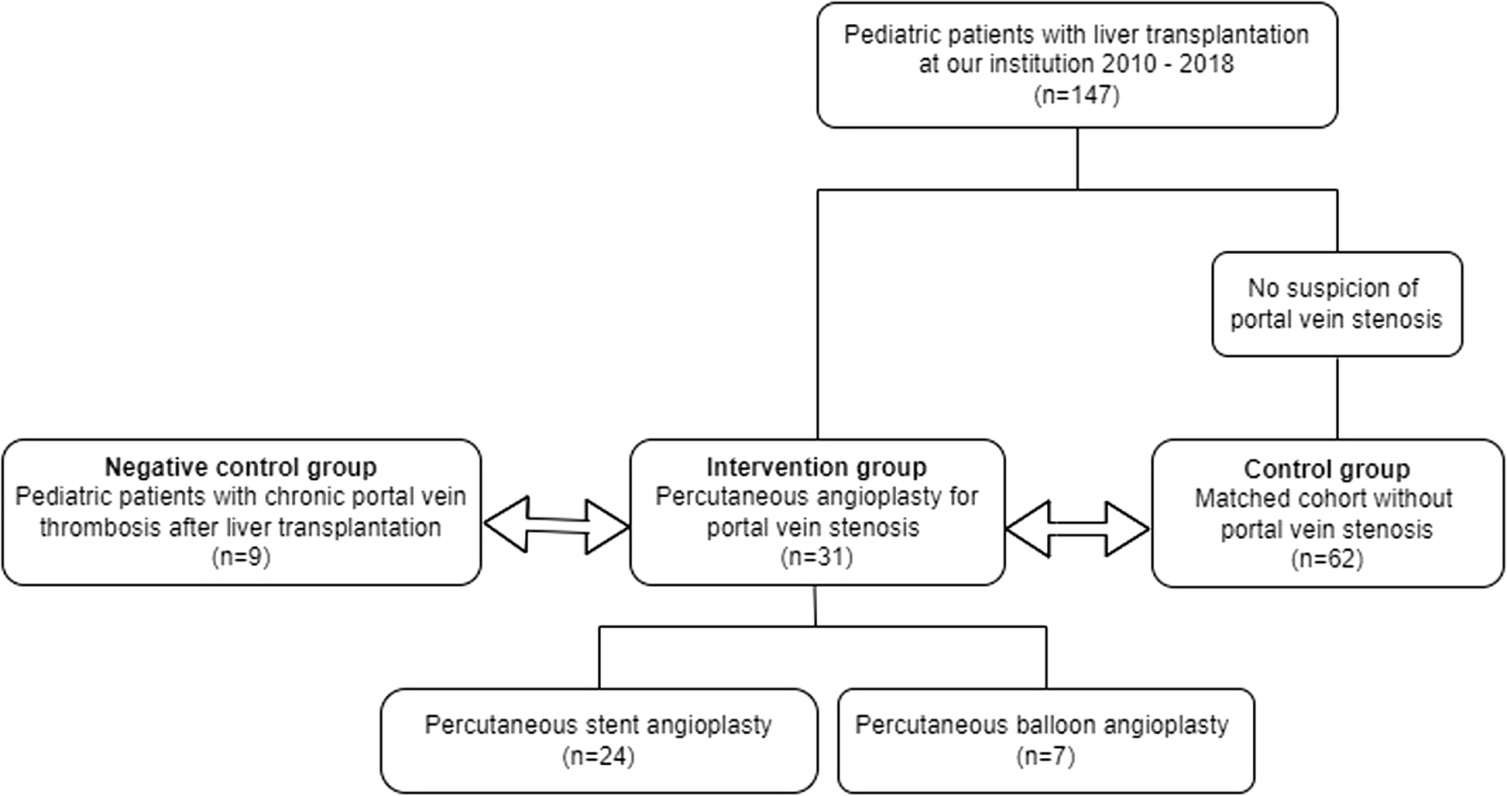
Flowchart of the study cohorts

This retrospective matched cohort study was undertaken at a pediatric liver transplant center. A retrospective review of the radiology information system was performed to identify patients under 18 years who underwent PTA (percutaneous transluminal balloon angioplasty [PTBA] or percutaneous transluminal stent angioplasty [PTSA]) of the PV after LT. Patients were assigned to the intervention group, if the LT was performed at our institution from 2010 to 2018 and if they underwent PTA for PVS at our pediatric interventional radiology center (Fig. 1).

A matched control group was selected (W. U. and A. D.) from patients without suspicion of PVS and endovascular intervention who underwent LT at our institution based on the time of LT (2010–2018) and age at transplantation (< 84 months). An additional negative control group comprised children with chronic PVT after LT. Due to the rarity of this condition at our center, the inclusion criteria for the negative control group were expanded to include children transplanted outside our institution and study period.

### Technique of endovascular treatment

All procedures were performed under general anesthesia. The peripheral PV or splenic vein was accessed via a sonographic guided transhepatic or transsplenic puncture. Four French (F)–6 F sheaths were placed and angulated 4 F or 5 F catheters were used to advance a microcatheter beyond the narrowing. For PTBA, 3.5 to 9 mm balloons were used (Mustang and Sterling balloon catheter, Boston Scientific, Marlborough, MA, USA). In cases of elastic recoil of the stenosis following PTBA, an oversized self-expandable stent was placed to permit repeat PTBA if needed. The stents placed for PTSA were 6 mm- or 7 mm-diameter Astron pulsar (Biotronik, Berlin, Germany), Sinus superflex (Optimed, Ettlingen, Germany), and Epic (Boston Scientific, Marlborough, MA, USA). The transsplenic and transhepatic puncture tracts were closed using gelfoam [8]. Immediately after intervention, patients underwent systemic anticoagulation with intravenous administration of heparin for 24–48 h (200–400 IU/kg/day, partial thromboplastin time target 50–60 s). Afterwards, low-molecular-weight heparin was administered subcutaneously once daily at a prophylactic dose for a period of 4 to 12 weeks postinterventional (depending on the individual risk assessment for PVT).

### Data collection and follow-up protocol

Data were collected (A. D., who worked under the supervision of W. U.) from electronic patient charts, the radiology information system, and the picture archiving computer system.

Clinical, laboratory, and sonographic parameters were monitored during follow-up (f/u) visits in our pediatric liver transplant clinic according to the local pediatric LT protocol (3, 6, and 12 months and then yearly after LT) which also included assessment for symptoms of PH due to PVC. Laboratory examinations included comprehensive blood tests assessing liver function including glutamate oxalacetate transaminase (GOT), glutamate pyruvate transaminase (GPT), gamma-glutamyl transferase (GGT), and bilirubin levels. Platelet count was used for evaluating hypersplenism.

### Study endpoints and definitions

The primary endpoint of this study was the assessment of graft and patient survival in the intervention group in comparison to the control group. In addition, graft and patient survival were compared to the negative control group. Graft loss was defined as the need for retransplantation or recipient death due to graft failure.

Secondary endpoints included (1) comparison of the posttreatment liver function and clinical outcomes with those of the control and negative control group; (2) assessment of the sequelae of PH based on platelet count and gastrointestinal varices/bleeding; and (3) evaluation of treatment-associated complications and long-term patency.

Technical success of PTA was defined as no or insignificant residual stenosis (< 30%) and improvement of intrahepatic PV flow/reduction of collateral flow after PTA. Long-term patency was defined as no need for reintervention during f/u. Complications were recorded according to the Cardiovascular and Interventional Radiological Society of Europe (CIRSE) [9].

### Statistical analysis

Summary statistics were performed to ensure comparability of the groups. Continuous variables are presented as median (interquartile range [IQR]) and were compared between groups using the non-parametric Mann–Whitney *U* test. Categorical data are presented as absolute and relative frequencies and were compared using the chi-square test of independence. Laboratory values at each visit (platelet count, GOT, GPT, GGT, and bilirubin) were compared between groups by using linear mixed models (repeated factor: time, subject: patient, covariance structure: autoregressive). Estimated marginal means with corresponding 95% confidence intervals (CI) are reported as effect estimates. As negative values for laboratory values are not physiologically plausible, the lower bounds of the 95% CIs were truncated at zero to prevent misleading interpretation of the results. Kaplan–Meier plots and log-rank tests were used to compare graft and patient survival. A *p* value < 0.05 was considered statistically significant. All analyses were performed using R (version 4.4.1, R Foundation for Statistical Computing, Vienna, Austria) and SAS (version 9.4, SAS Institute, Cary, NC).

## Results

### Patient characteristics

Between 2010 and 2018, a total of 147 pediatric patients received liver transplants at our institution. Thirty-one out of 147 patients (21%) underwent PTA for PVS (intervention group: 19 female). Median age at time of LT was 5.8 months (IQR 4.9–10.7 months) in the intervention group. A matched cohort group with 62 patients (24 female; median age at time of LT 7.6 months, IQR 5.6–25.4 months) was selected from children who were transplanted at our institution between 2010 and 2018 and did not present with PVC. Additionally, a negative control group with 9 patients (2 female; median age at time of LT 17.4 months, IQR 3.5–25.7 months), who were transplanted between 2003 and 2017, was identified (Fig. 1). Demographic and clinical data of the intervention, matched control, and negative control groups are listed in Table 1. Median f/u time was 67 months (IQR 36–82 months) in the intervention group, 42 months (IQR 25–74 months) in the control group, and 85 months (IQR 24–137 months) in the negative control group.

**Table 1:** Demographic and clinical data of children with PVS (intervention group), without PVS (matched control group), and chronic PVT (negative control group)

Characteristic	Intervention group^a^ (***n*** = 31)	Matched control group^a^ (***n*** = 62)	***p*** value^b^	Negative control group (***n*** = 9)	***p*** value^c^	***p*** value^d^
Sex			**0.040**		**0.039**	0.337
Male	12 (39)	38 (61)		7 (78)		
Female	19 (61)	24 (39)		2 (22)		
Age (months)	5.8 [4.9–10.7]	7.6 [5.6–25.4]	0.059	17.4 [3.5–25.7]	0.319	0.916
Body weight (kg)	6.6 [5.5–8.2]	8.3 [6.2–10.2]	**0.027**	9.0 [5.5–11.3]	0.302	0.951
Body height (cm)	65 [62–73]	70 [63–85]	**0.046**	79 [58–86]	0.281	0.921
Indication for LT			0.481		**0.010**	**0.012**
Biliary atresia	26 (84)	46 (74)		3 (33)		
Acute liver failure	2 (6)	4 (6)		1 (11)		
Metabolic disease	3 (10)	3 (5)		4 (44)		
Others	0	9 (15)		1 (11)		
Donor			0.652		0.279	0.387
DDLT	11 (35)	25 (40)		5 (56)		
LDLT	20 (65)	37 (60)		4 (44)		
Type of transplant			0.081		0.203	0.700
Left lateral split	27 (87)	58 (94)		8 (89)		
Right split	3 (10)	1 (2)		0		
Whole organ	0	3 (4)		1 (11)		
Auxiliary left lateral split	1 (3)	0		0		
Graft weight (g)	283 [249–339]	275 [236–355]	0.700	242 [185–271]	0.076	0.098
Graft/patient body weight ratio (%)	4.4 [3.5–5.2]	3.4 [2.7–4.2]	**0.008**	2.4 [2.0–2.9]	**0.010**	0.184

Data are shown as absolute numbers (%) or median [interquartile range]

*DDLT* deceased donor liver transplantation, *LDLT* living donor liver transplantation, *LT* liver transplantation

^a^The two groups were matched for age and time of LT (2010–2018)

^b^*p* value for comparison of intervention group and matched control group

^c^*p* value for comparison of intervention group and negative control group

^d^*p* value for comparison of control group and negative control group

### Percutaneous angioplasty (technical success, complications, long-term patency)

**Fig. 2:**
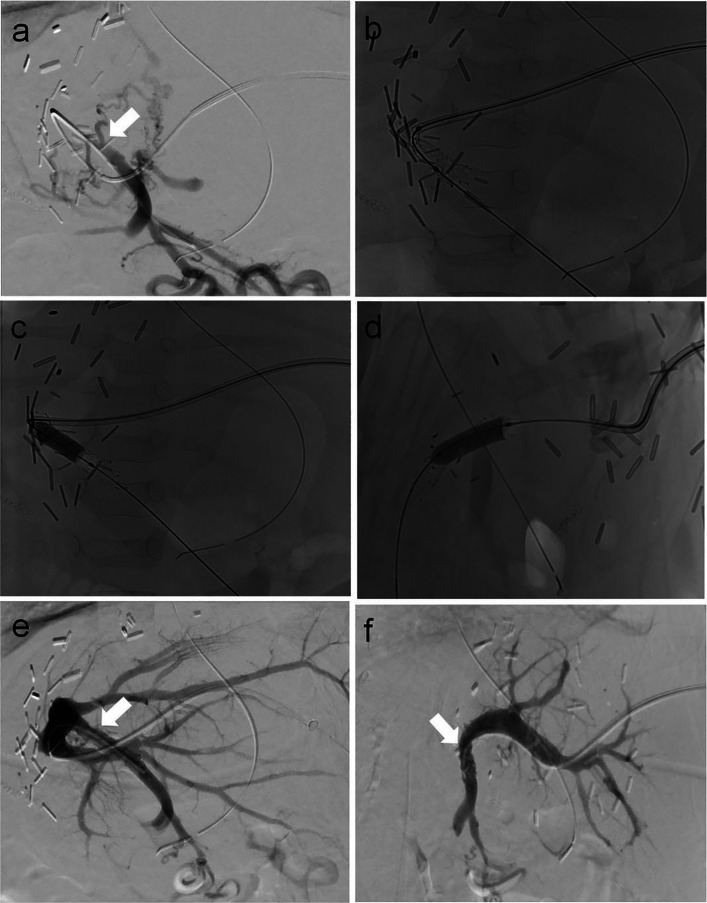
Percutaneous transluminal stent angioplasty (PTSA) for portal vein stenosis (PVS) via transhepatic access in a 5-months-old male patient, 4 weeks post left lateral split-liver transplantation. **a** Digital subtraction angiography (DSA) via a 4 F sheath and a 4 F catheter demonstrates complete portal vein occlusion with filling of collateral veins. **b**–**d** Fluoroscopic images following stent placement (Astron pulsar 18, 7/20 mm) and balloon angioplasty. **e** and **f** Post-PTSA DSA in two planes reveals complete resolution of the PVS

Median time between LT and PTA was 3.9 months (IQR 1.6–7.7 months). A transhepatic access for PTA was used in 28/31 (90%) patients, a transsplenic access in 2/31 (6%) patients, and both routes in 1/31 (4%) patient. In 24/31 (77%) cases, PVS was treated by PTSA (Fig. 2); 7/31 (23%) patients were treated with PTBA alone. There was one (1/31 [3%]) procedure-related complication: one patient showed a perihepatic hematoma in postinterventional sonography, which resolved without treatment (CIRSE complication grade 2). Technical success was achieved in 30/31 (97%) cases. One patient presented with a sonographic PVS of 60% 3 months after PTBA without clinical signs of PH and progression and no need for reintervention during f/u (104 months). One patient underwent endovascular treatment for PVS with associated thrombosis. Residual peripheral thrombi persisted in f/u angiography, while the central PVS resolved and did not recur until the end of f/u (108 months). Long-term PV patency after PTA until the end of f/u was 100%.

### Graft and patient survival

**Fig. 3:**
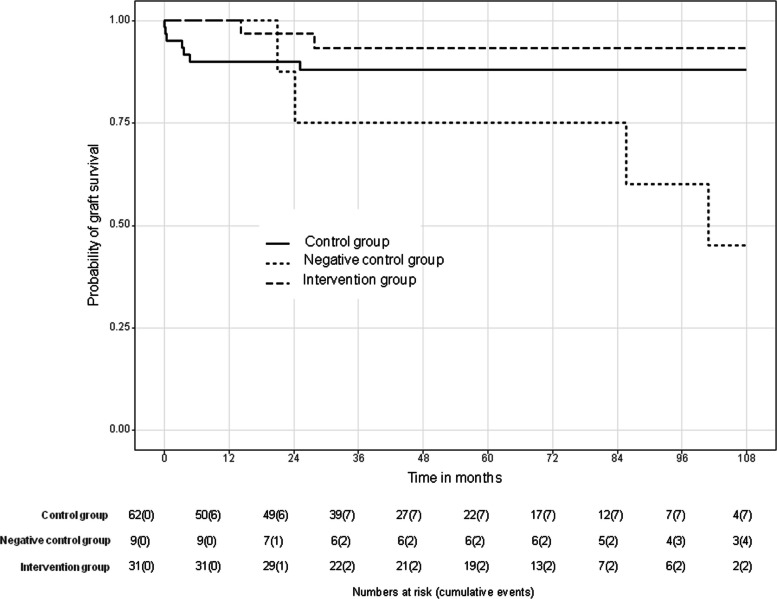
Death-censored Kaplan–Meier curves of graft survival in 31 pediatric patients after PTA for PVS compared with a matched control group (62 patients) and a negative control group (9 patients with untreated PVT)

Graft survival was comparable between the intervention and control group (*p* = 0.380), with 7-year survival rates of 93% (95% CI: 84%, 100%) and 88% (95% CI: 80%, 97%), respectively. Graft survival was significantly higher in the intervention group compared to the negative control group (*p* = 0.032) with 7-year graft survival of 75% (95% CI: 50%, 100%) in the negative control group (Fig. 3). Graft loss occurred in 2/31 cases in the intervention group (secondary sclerosing cholangitis and chronic graft rejection) and in 7/62 in the control group (chronic graft rejection [1/7], acute graft failure [2/7], chronic cholangitis [1/7], secondary sclerosing cholangitis [1/7], occlusion of the hepatic artery [2/7]). In the negative control group, graft loss occurred in 4/9 cases: 3/9 patients were retransplanted due to graft failure (chronic PVT 2/3 and additional chronic cholangitis 1/3). 1/9 patient died due to graft failure (chronic PVT and secondary sclerosing cholangitis).

**Fig. 4:**
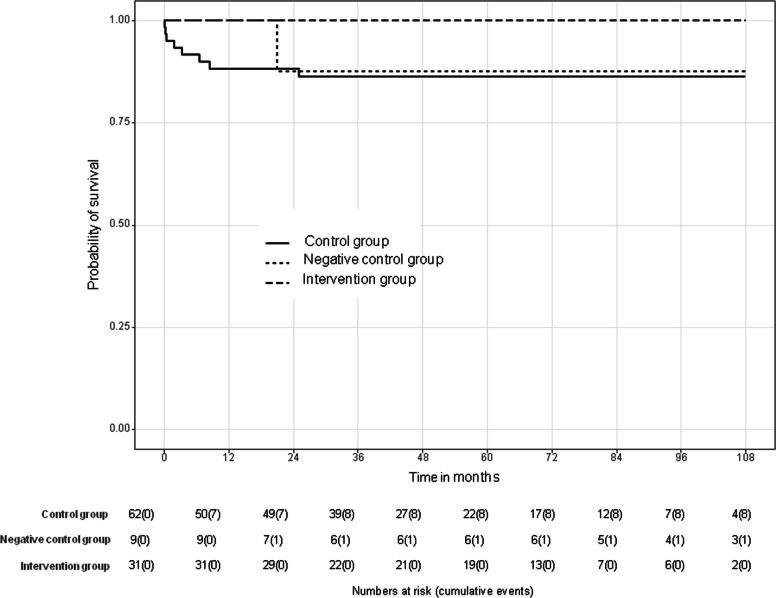
Kaplan–Meier curves of patient survival in 31 pediatric patients after PTA for PVS compared with a matched control group (62 patients) and a negative control group (9 patients with untreated PVT)

Patient survival was higher in the intervention group in comparison to the control group (*p* = 0.034) with 100% (95% CI: 100%, 100%) 7-year patient survival in the intervention group versus 86% (95% CI: 78%, 96%) in the control group. In comparison to the intervention group, patient survival was reduced in the negative control group approaching statistical significance (*p* = 0.053, Fig. 4). Seven-year patient survival in the negative control group was 88% (95% CI: 67%, 100%).

No patient of the intervention group died. In the control group, a total of 8/62 patients died, the majority with a functioning graft (cardiovascular failure [3/8], severe systemic inflammatory response syndrome [2/8], post-transplant lymphoproliferative disorder [1/8], acute graft failure [2/8]). In the negative control group, 1/9 patient died due to secondary sclerosing cholangitis and chronic graft failure due to PVT.

### Clinical outcomes

#### Course of platelet counts in the intervention group

Platelet counts increased significantly after endovascular treatment: 3 months postinterventional + 48/nL (95% CI: + 77/nL, + 20/nL; *p* = 0.002), 6 months postinterventional + 72/nL (95% CI: + 96/nL, + 48/nL; *p* < 0.001), 12 months postinterventional + 51/nL (95% CI: + 92/nL, + 10/nL; *p* = 0.016), and 24 months postinterventional + 84/nL (95% CI: + 115/nL, + 52/nL; *p* < 0.001).

#### Comparison of liver function and hypersplenism during follow-up

The course of the mean values of the laboratory markers for liver function (GOT, GPT, GGT, bilirubin) and hypersplenism (platelet counts) were similar in the intervention group and the control group during long-term f/u (Table 2).

**Table 2:** Comparison of laboratory parameters for liver function and hypersplenism in the intervention group and the control group 3, 4, and 5 years after liver transplantation using linear mixed models

Parameter	No. of patients	Intervention group	No. of patients	Control group	Difference Intervention group/control group	***p*** value
		Estimated mean (95% CI)		Estimated mean (95% CI)	Estimated mean (95% CI)	
Platelet count (counts/nL)						
3 years after LT	26	294 (255, 333)	42	256 (226, 286)	38 (87, 10)	0.126
4 years after LT	19	300 (256, 343)	30	290 (256, 325)	10 (65, − 46)	0.740
5 years after LT	19	284 (239, 329)	24	274 (235, 313)	10 (69, − 50)	0.746
GOT (U/L)						
3 years after LT	26	39 (0, 102)	40	39 (0, 90)	0 (80, − 81)	0.996
4 years after LT	19	36 (0, 110)	30	36 (0, 94)	0 (94, − 94)	0.999
5 years after LT	18	35 (0, 109)	23	38 (0, 105)	3 (97, − 104)	0.945
GPT (U/L)						
3 years after LT	26	30 (0, 72)	42	28 (0, 61)	2 (55, − 52)	0.956
4 years after LT	19	31 (0, 80)	30	26 (0, 65)	5 (67, − 58)	0.878
5 years after LT	19	29 (0, 78)	24	30 (0, 74)	1 (64, − 67)	0.975
GGT (U/L)						
3 years after LT	26	46 (0, 121)	42	39 (0, 97)	8 (102, − 87)	0.876
4 years after LT	19	48 (0, 135)	30	37 (0, 106)	11 (122, − 100)	0.845
5 years after LT	18	48 (0, 137)	24	32 (0, 110)	15 (133, − 102)	0.799
Bilirubin (mg/dL)						
3 years after LT	26	0.4 (0, 1.8)	41	0.5 (0, 1.6)	− 0.1 (1.7, − 1.9)	0.880
4 years after LT	19	0.5 (0, 2.1)	30	0.6 (0, 1.9)	− 0.1 (2.0, − 2.2)	0.938
5 years after LT	19	0.5 (0, 2.1)	23	0.4 (0, 1.9)	0.1 (2.3, − 2.1)	0.955

*CI* confidence interval, *GGT* gamma-glutamyl transferase, *GOT* glutamate oxalacetate transaminase, *GPT* glutamate pyruvate transaminase, *LT* liver transplantation

#### Gastroesophageal varices

Four out of thirty-one (12.9%) patients in the intervention group presented with gastroesophageal varices. After PTA (in 3/4 cases additional endovascular coiling of varices during PTA), all varices decreased and no gastrointestinal bleeding occurred until the end of f/u. In the control group, no gastroesophageal varices/bleeding was documented during f/u. In the negative control group, 8/9 (89%) patients presented with gastroesophageal varices and 2/8 (25%) with additional bleeding. One out of eight (12.5%) patients was treated endoscopically during f/u; in 7/8 (87.5%) patients, gastroesophageal varices persisted until the end of f/u.

## Discussion

Seven-year patient survival after PTA for PVS after pediatric LT under the age of 7 years was with 100% (95% CI: 100%, 100%) higher in comparison to a matched cohort of pediatric patients after LT without PVS (86%, 95% CI: 78%, 96%). Seven-year graft survival was similar with 93% (95% CI: 84%, 100%) in the intervention group and 88% (95% CI: 80%, 97%) in the control group. Patients with chronic PVT showed a significantly lower graft survival in comparison to the intervention group underlining the relevance of treating PVT. There was an improvement of signs of PH in the intervention group and the course of laboratory parameters during long-term f/u were similar to those of the cohort without PVS indicating clinical success of PTA. There were no major procedure-related complications and the minor complication rate was low (3%). Long-term patency rate was 100%.

Age matching was employed to reduce effects related to anatomic size variations. Matching for time period of transplantation was conducted to minimize confounding effects arising from variations in surgical techniques and operating personnel. However, regarding the patient characteristics, the median graft/patient body weight ratio was significantly higher in the intervention group (4.5%) in comparison to the control group (3.4%, *p* = 0.008), which aligns with the results of Ueda et al. [10]. They have found a low body weight (< 6 kg) and a graft/patient body weight ratio > 4% to be risk factors for PVC in pediatric patients.

Our low complication and high long-term patency rates of PTA for PVS are consistent with previous studies [4, 11, 12] and due to its effectiveness, safety, and minimally invasive approach interventional revascularization has become the preferred treatment option for PVS in children [5]. The observed outcomes in patient and graft survival provide additional support for the efficacy of this strategy. Graft survival was markedly lower in the negative control group directly reflecting the natural progression of PVS in the absence of successful therapeutic management. PVT has been reported to be detrimental to both patient and graft survival after LT with a higher risk in pediatric patients most likely due to the small size of the recipient and donor vein [13]. Morbidity from PH due to PVS represents an additional clinical burden beyond graft loss. This is evidenced by the negative control group with chronic PVT, where gastrointestinal varices and variceal bleeding were observed in 89% of cases, with a recurrence rate of 25%. The severity of these complications often correlates with the degree of PH and may necessitate intensified medical monitoring and is probably underestimated when only focusing on graft survival. There are few studies in the literature with small sample sizes about interventional treatment of pediatric chronic PVT resulting in total occlusion of the PV [11, 14–18]. Recently Dulcetta et al. reported about five post-transplant children (youngest age 7 years) with chronic total PV occlusion, whose portal flow was restored by percutaneous revascularization procedures [14]. Beyond these encouraging results, we suggest that the early treatment of PV stenosis may have a prophylactic effect, potentially preventing thrombosis, minimizing technically demanding interventions and subsequent morbidity.

To date, there is no consensus on the most optimal treatment strategy, particularly regarding stent placement. The review by Kyaw et al., including 213 pediatric patients, suggested that PTBA is an appropriate initial treatment, with stent placement reserved for selected cases or as a salvage intervention [19]. Messana et al. also concluded in their recent study that PTBA should be the preferred first-line treatment for PVS in pediatric liver transplant recipients, with stent placement reserved for cases unresponsive to balloon angioplasty. However, in their study, recurrent stenosis occurred in 6/21 patients after PTBA necessitating one or more additional procedures subsequently [7]. At our institution, most cases (24/31, 77%) were treated with PTSA resulting in a high patency rate without need for reintervention as opposed to PTBA alone, which aligns with the results of previous studies [20, 21]. Formerly, in the early stages of interventional treatment of PVS, it has been suggested that PTBA should be preferred over PTSA in children due to concerns about stent migration, potential technical problems in case of retransplantation, and the “fixed stenosis” phenomenon [4, 22]. It has been theorized that a metal stent may lead to long-term complications in terms of a relative stenosis, as the stent does not grow with the child [23, 24]. These early concerns have not been confirmed and the systematic review of Sare et al. including 243 percutaneous interventions for PVS found PTSA the superior technique due to lower reintervention rates [12]. Bukova et al. also suggest primary PTSA as first-line treatment for PVS, especially to avoid early restenosis in small children potentially necessitating multiple reinterventions [20]. Based on our study protocol and the results obtained, we recommend stent placement in the same session in case of recoiling following PTBA and anticoagulant therapy with intravenous heparin and low-molecular-weight heparin. Postinterventional anticoagulation, which may impact patency rates, is still debated in the literature. Alfares et al. conclude in their review analyzing 22 cohort studies with 362 pediatric patients that the current data are not sufficient to recommend any specific anticoagulation therapy over another [5]. Bukova et al. who analyzed a comparable study cohort after PTSA report about a primary patency rate of 77% at 7 years with dual antiplatelet therapy (acetylsalicylic acid and clopidogrel).

This was a retrospective study and matching for time of transplantation and age was performed to control bias due to child growth and treatment changes over time. The matched control group had a median age of 7.6 months, compared to 5.8 months in the intervention group. Although this difference was not statistically significant, the potential influence of patient age as a risk factor for poorer patient and graft survival cannot be excluded. Small cohort sizes prevented matching for additional covariates such as diagnosis, graft-to-body weight ratio, or graft type, limiting the validity of the case–control matching. Furthermore, comparison of PTBA and PTSA was not reasonably feasible due to the limited number of PTBA alone. The planned PORTAL (Portal vein Obstruction Revascularisation Therapy After Liver transplantation) registry multicenter project with its aim to assess the prevalence, current management practices, and efficacy of treatment will certainly provide valuable information on this topic [6].

In conclusion, 7-year graft and patient survival after PTA for PVS were comparable to that of patients without PVS after pediatric LT. Graft survival was lower for patients with chronic PVT. Clinical course and liver function after PTA were comparable to patients without PVS. In combination with a low complication and high long-term patency rate, the value of PTA in the treatment of PVS is reinforced by our results.

## Data Availability

The datasets used and analyzed during the current study are available from the corresponding author on reasonable request.

## Abbreviations

CIRSE: Cardiovascular and Interventional Radiological Society of Europe
f/u: Follow-up
GGT: Gamma-glutamyl transferase
GOT: Glutamate oxalacetate transaminase
GPT: Glutamate pyruvate transaminase
IQR: Interquartile range
LT: Liver transplantation
PH: Portal hypertension
PTA: Percutaneous angioplasty
PTBA: Percutaneous transluminal balloon angioplasty
PTSA: Percutaneous transluminal stent angioplasty
PVC: Portal vein complications
PVS: Portal vein stenosis
PVT: Portal vein thrombosis
